# Artifact reduction in contrast-enhanced mammography

**DOI:** 10.1186/s13244-022-01211-w

**Published:** 2022-05-13

**Authors:** Gisella Gennaro, Enrica Baldan, Elisabetta Bezzon, Francesca Caumo

**Affiliations:** Breast Imaging Unit, Veneto Institute of Oncology (IOV), IRCCS. Via Gattamelata 64, 35128 Padua, Italy

**Keywords:** Mammography, Contrast agent, Artifact

## Abstract

**Objective:**

To evaluate the effectiveness of a new algorithm developed to reduce artifacts in dual-energy subtraction (DES) contrast-enhanced mammography (CEM) images while preserving contrast enhancement of possible lesions.

**Methods:**

A retrospective multi-reader paired study was performed by using 134 CEM studies obtained from the first 134 women enrolled in a prospective clinical study aiming to compare the clinical performance of CEM to those of breast MRI in screening of women at increased risk of breast cancer. Four experienced readers compared independently the standard (STD) DES images with those obtained by reprocessing the raw images by a new algorithm (NEW), expected to reduce the DES artifact intensity. The intensity of three types of artifacts (breast-in-breast, ripple, and skinfold enhancement) and the intensity of possible contrast uptake were assessed visually and rated using a categorical ordinal scale. Proportions of images rated by the majority of readers as “Absent”, “Weak”, “Medium”, “Strong” in each artifact intensity category were compared between the two algorithms. P-values lower than 0.05 were considered statistically significant.

**Results:**

The NEW algorithm succeeded in eliminating 84.5% of breast-in-breast artifacts, 84.2% of ripple artifacts, and 56.9% of skinfold enhancement artifacts versus STD DES images, and reduced the artifact intensity in 12.1%, 13.0%, and 28.8% of the images, respectively. The visibility of lesion contrast uptake was the same with the STD and the NEW algorithms.

**Conclusion:**

The new dual-energy subtraction algorithm demonstrated to be effective in reducing/eliminating CEM-related artifacts while preserving lesion contrast enhancement.

## Key Points

- Most DES images obtained by STD algorithm present breast-in-breast, ripple, and skinfold artifacts.
- The NEW DES algorithm removes/reduces breast-in-breast artifacts (84.5% /12.1%).
- The NEW DES algorithm removes/reduces ripple artifacts (84.2% /13.0%).
- The NEW DES algorithm removes/reduces skinfold artifacts (56.9% /28.8%).
- The NEW DES algorithm preserves lesion contrast uptake.

## Background

Contrast-enhanced mammography (CEM) is a dual energy imaging technique consisting of the acquisition of an image pair for each mammography view starting a couple of minutes after the administration of an iodinated contrast agent. The low-energy (LE) image has been shown to be equivalent to a standard mammogram, as it is obtained with a mammographic spectrum whose photon energies are below the 33.2 keV k-edge of iodine, and thus, the contrast agent is not apparent in the image. High-energy image (HE) acquisition is performed by increasing photon energies above the 33.2 keV k-edge of iodine and this image is recombined with the LE-image to obtain a “dual-energy subtraction” (DES) image depicting the contrast uptake. The HE-image is nondiagnostic [1, 2]. Contrast-enhanced mammography is currently used as problem solving tool to address inconclusive findings at mammography, for staging of breast cancer, in workup of symptomatic patients; moreover, CEM has proven its potentials as alternative to breast MRI for neoadjuvant response monitoring, in screening of women with dense breasts, and in screening of women at increased risk for breast cancers [1, 3–5].

As any other imaging technique, CEM is affected by some artifacts that have been described in a few pictorial articles [6, 7]. In a very recent paper, Neppalli et al. reported that some DES CEM images artifacts are manufacturer dependent; in other words, technological differences between equipment and differences in subtraction algorithms can determine manufacturer-specific artifacts in DES CEM images [8].

One of the most frequent CEM-related artifact is the “breast-in-breast” artifact (also reported as “halo” or “rim” artifact) caused by non-uniform breast thickness and scattered radiation, and resulting in a C-shaped “halo” of apparent enhancement within the breast periphery in the subtraction image. Another common artifact in CEM imaging is the “ripple” artifact (also reported as “motion” or “misregistration” artifact) caused by a misregistration between LE- and HE-images likely due to a slight patient motion, resulting in faint alternating black and white lines in the subtraction image. In a retrospective analysis by Yagil et al., it was concluded that, despite the high probability of artifact occurrence, none of the artifacts had interfered with image interpretation [9].

However, in medical imaging image quality should be optimized for the clinical task [10, 11], and how much the artifact presence might or might not affect CEM interpretation depends on the clinical tasks and the purpose for which the CEM exam was performed. Whenever CEM is used as workup imaging of known lesions, it is understandable that the artifacts described above would not hamper the image interpretation; on the opposite, if CEM would be prospectively used as a screening tool in specific target populations, artifact presence might limit the detectability of small lesions. For this reason manufacturers are proposing artifact reduction algorithms in order to reduce artifact impact on image interpretation while improving CEM image quality [12].

In this study, we evaluated the effectiveness of a new algorithm developed to reduce artifacts in dual-energy subtraction CEM images while preserving contrast enhancement by possible lesions.

## Materials and methods

The study population consisted of 134 consecutive CEM studies obtained by the first 134 women enrolled in a prospective clinical study (CE IOV #2017/92) aiming to compare the clinical performance of CEM to those of breast MRI in screening of women at increased risk of breast cancer. Risk assessment was performed by the Tyrer-Cuzick model [13, 14]. Intermediate and high risk women were identified applying respectively 17% and 30% threshold values to the lifetime risk resulting from the risk model [15]. The study was approved by the Institutional Ethics Committee. Informed consent was signed by all women enrolled in the clinical study. Two-view (cranio-caudal—CC, and medio-lateral oblique—MLO) bilateral CEM exams were performed by a GE Senographe Pristina unit (GE Healthcare, Chicago, IL), starting two minutes after injection of 1.5 mL/kg iodinated contrast agent (GE Omnipaque 350) by an automatic injector (3 mL/s). The CEM algorithm automatically applies a standard post-processing to unprocessed LE-images for their interpretation alongside DES images that enhance possible contrast uptake by recombining unprocessed LE- and HE-images of each mammography view. For the purposes of this study, LE- and HE-images were reprocessed twice, the first time to get the standard (STD) DES images, the second one to obtain DES images by a new algorithm (NEW), expected to reduce the artifact intensity. Processing of the LE-images was unchanged with the two algorithms, and was not considered in the present study.

In order to evaluate the effectiveness of the new DES algorithm which was expected to reduce both breast-in-breast and ripple artifacts, and to attenuate the visibility of possible skinfolds while preserving enhancement due to contrast uptake, four experienced readers (all with more than 10 years of experience in breast imaging) were asked to assess for each view and each algorithm the intensity of breast-in-breast and of ripple artifacts, as well as the intensity of possible skinfold enhancement, and/or possible contrast uptake, using a four-class ordinal scale: 1 = Absent/None, 2 = Weak, 3 = Medium, 4 = Strong.

STD and NEW subtraction images for each view (RCC, LCC, RMLO, LMLO) were presented side-by-side on the two 5 MP displays of a review workstation (GE SenoIris), and each reader performed the artifact assessment independently. At the end of the per-view evaluation, all views processed with the two algorithms were shown side-by-side, and the readers were asked to compare the two algorithms using a Likert scale: − 2 = NEW is much worse than STD, − 1 = NEW is worse than STD, 3 = NEW is equal to STD, + 1 = NEW is better than STD, + 2 = NEW is much better than STD.

The score by the majority of readers obtained by calculating the statistical mode from the individual scores assigned to each image in each dataset was used to evaluate the capability of the NEW algorithm of eliminating or reducing each type of artifact compared to the STD algorithm. The probability of occurrence with the STD algorithm, the capability of the NEW algorithm of completely removing or reducing each type of artifact were calculated, as well as the image fraction for which the NEW algorithm did not reduce the artifacts.

Moreover, the proportions of images scored by the majority of readers in the same category were compared between the two algorithms for each of the three artifact types considered, and for the contrast uptake visibility, using a Chi-Square test. P-values lower than 0.05 were considered statistically significant.

The level of agreement across the four readers was determined by calculating the Fleiss’ kappa from the scores assigned to the intensity of each artifact type and to the contrast uptake visibility by the four readers to the images processed by the two algorithms.

Finally, a descriptive analysis of the overall preferences by the majority of readers between the two algorithms was performed. Statistical analysis was carried out using R version 4.1.1 and OriginPro 2020b.

## Results

The study population included 134 women at increased risk for breast cancer whose characteristics are reported in Table 1.

**Table 1:** Characteristics of women included in the study population: number, age, menopausal state, breast density, and risk category

Characteristics	Values
No. of women	
Bilateral exam	129
Unilateral exam	5
Total	134
No. of images	
CC views	263
MLO views	263
Total	526
Age (y)	
Mean ± SD	49.9 ± 9.2
Range	(35, 73)
Menopausal state	
Premenopausal	67 (50.0%)
Perimenopausal	21 (15.7%)
Postmenopausal	46 (34.3%)
Breast Density*	
BIRADS a	13 (9.7%)
BIRADS b	19 (14.2%)
BIRADS c	49 (36.6%)
BIRADS d	53 (39.6%)
Risk category**	
High	97 (72.4%)
Intermediate	37 (27.6%)

*Breast density was assessed according to the 5th edition of Breast-Imaging Reporting and Data System (BIRADS) by the American College of Radiology

**High risk women: if lifetime risk calculated by the Tyrer-Cuzick risk model is above 30%. Intermediate risk women: if lifetime risk calculated by the Tyrer-Cuzick risk model is between 17 and 30%

The total number of evaluated views was 526 resulting from 129 bilateral and 5 unilateral CEM exams, equally distributed in CC and MLO views. The mean age was 49.9 ± 9.2 years, ranging between 35 and 73 years. Premenopausal women were 50.0% (67/134), while the remaining 50.0% were either perimenopausal (21/134 = 15.7%) or postmenopausal (46/134 = 34.3%). Women included in the study had mostly dense breasts: 49/134 (36.6%) categorized as BIRADS c and 53/134 (39.6%) as BIRADS d against 13/134 (9.7%) categorized as BIRADS a and 19 (14.2%) as BIRADS b. They were predominantly high-risk women (72.4% = 97/134), while the remaining 27.6% (37/134) were at intermediate risk.

As previously mentioned, the image scores by the majority of readers were used to compare the artifact frequency between the two algorithms. The number and the proportion of images affected by artifacts with the STD algorithm, and the number and the proportion of images for which the NEW algorithm either completely removed or at least reduced the artifact intensity obtained using the scores by the majority of readers are provided in Table 2 for each artifact type.

**Table 2:** Number and proportion of images affected by artifacts with the STD algorithm, number and proportion of images for which the NEW algorithm either completely removed or at least reduced the artifact intensity obtained using the scores by the majority of readers, and number and proportion of images for which the NEW algorithm did not correct the artifact

	Artifact type					
	Breast-in-Breast		Ripple		Skinfold	
	No. of images	Proportion (%)	No. of images	Proportion (%)	No. of images	Proportion (%)
Artifact occurrence with the STD algorithm	412/526	78.3	285/526	54.2	288/526	54.8
CC	227/412	55.1	114/285	40.0	115/288	39.9
MLO	185/412	44.9	171/285	60.0	173/288	60.1
Artifact removal by the NEW algorithm	348/412	84.5	240/285	84.2	164/288	56.9
Artifact reduction by the NEW algorithm	50/412	12.1	37/285	13.0	83/288	28.8
Artifact reduction failure by the NEW algorithm	14/412	3.4	8/285	2.8	41/288	14.2

Results for the three types of artifacts: breast-in-breast, ripple, and skinfold enhancement

The breast-in-breast artifact occurred in 78.3% (412/526) of the DES CEM images and was prevalent in CC views (CC = 55.1% vs. MLO = 44.9%, *P* = 0.0034); the ripple artifact and the skinfold enhancement occurred in 54.2% and 54.8% of images, respectively, and were both prevalent in MLO views (ripple artifact: CC = 40.0%, MLO = 60.0%, *P* < 0.0001; skinfold enhancement artifact: CC = 39.9%, MLO = 60.1%, *P* < 0.0001). The NEW algorithm successfully eliminated 84.5% and reduced 12.1% of breast-in-breast artifacts; the results were respectively 84.2% and 13.0% for ripple artifacts and 56.9% and 28.8% of skinfold enhancement artifacts. The percentage of images for which the NEW algorithm did not reduce the artifacts was very small (3.4% for breast-in-breast, 2.8% for ripple, and 14.2% for skinfold enhancement artifacts), and in almost all those cases the artifact intensities were scored “Weak”.

**Fig. 1:**
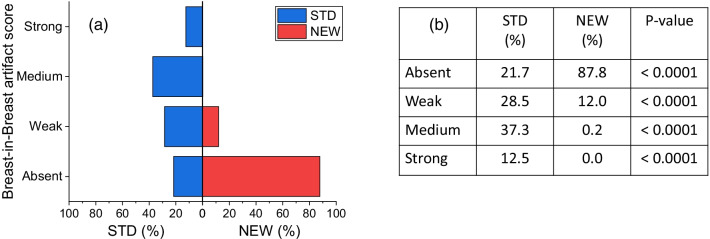
**a** Mirror bar graphs showing the percentages of DES CEM images for which the intensity of breast-in-breast artifact was scored “Absent”, “Weak”, “Medium” or “Strong” by the majority of readers, when the images were obtained with the STD (blue bars) and the NEW (red bars) algorithm, respectively; **b** results of comparison between proportions associated with STD and NEW algorithms for each category of breast-in-breast artifact and related p-values

Focusing on the most frequent breast-in-breast artifact, Fig. 1 shows the proportions of DES CEM images obtained with the two subtraction algorithms for each of the four categories scored by the majority of readers on the basis of the artifact intensity.

The NEW algorithm substantially removed the breast-in-breast artifact, significantly increasing the percentage of images classified as “artifact-free” (“Absent” score: 27.1% (STD) vs. 87.8% (NEW), *P* < 0.0001). The proportions of images showing “Weak”, “Medium” or “Strong” breast-in-breast artifact with the STD algorithm were all significantly reduced by the NEW algorithm, and the number of images processed by the NEW algorithm still showing a “Medium” or “Strong” artifact was very close to zero.

**Fig. 2:**
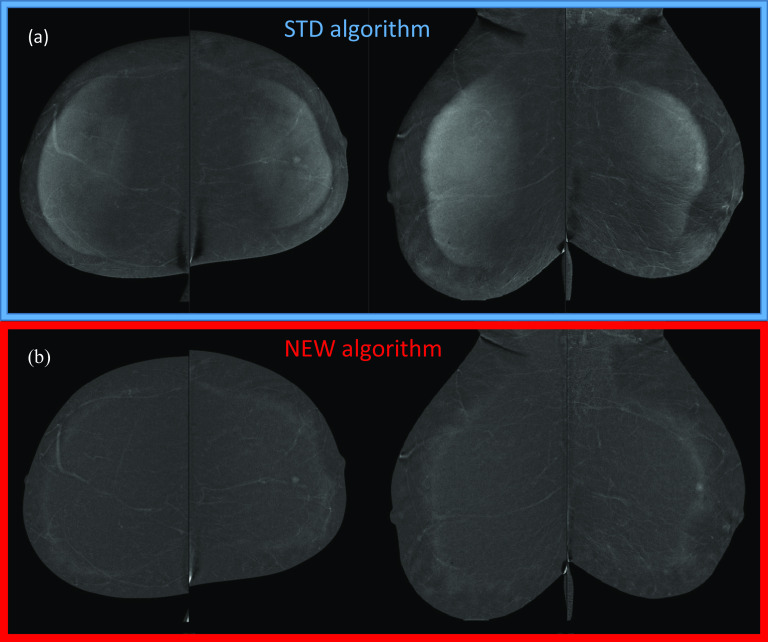
Postmenopausal woman 56 yo at high risk for breast cancer with large, fatty breasts. **a** DES CEM images obtained by the STD algorithm showing the breast-in-breast artifact in all the four views; **b** DES CEM images obtained by the NEW algorithm, where the artifact was completely removed

Figure 2a depicts an example of breast-in-breast artifact classified as “Strong” by the majority of readers in all the four DES CEM views obtained by the STD subtraction algorithm; in Fig. 2b the artifact was “cleaned” by the NEW algorithm.

**Fig. 3:**
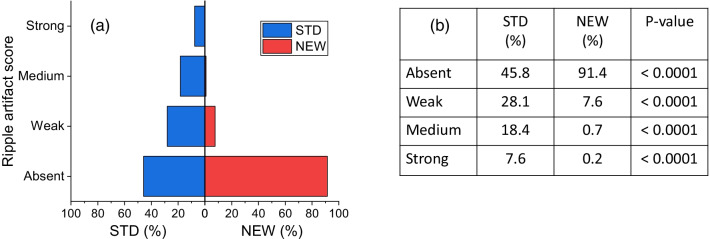
**a** Mirror bar graphs showing the percentages of DES CEM images for which the intensity of ripple artifact was scored “Absent”, “Weak”, “Medium” or “Strong” by the majority of readers, when the images were obtained with the STD (blue bars) and the NEW (red bars) algorithm, respectively; **b** results of comparison between proportions associated with STD and NEW algorithms for each category of ripple artifact and related p-values

Figure 3 shows the proportions of ripple artifacts scored by the majority of readers in the four categories when the DES images were processed with the STD and the NEW algorithm, and the related test results.

Despite the ripple artifact was present in more than half of the DES CEM images when they were obtained with the STD algorithm, the NEW algorithm almost completely eliminated this type of artifact (“Absent” score: 45.8% (STD) vs. 91.4% (NEW), *P* < 0.0001).

**Fig. 4:**
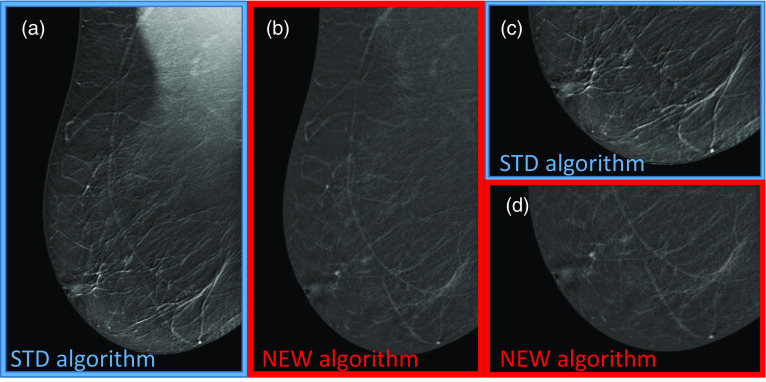
Premenopausal woman 46 yo at high risk for breast cancer with large, fatty breasts. **a** RMLO DES CEM view obtained by the STD algorithm showing the ripple artifact in the lower part of the image; **b** RMLO CEM view obtained by the NEW algorithm, where the artifact was completely removed; **c** Detail of the ripple artifact in the lower part of the RMLO CEM view with the STD algorithm; **d** Same detail of the lower part of the RMLO CEM view after removal by the NEW algorithm

Figure 4 shows an example of ripple artifact in the lower part of the RMLO view, that can be recognized by the presence of “linear stripes” with the STD algorithm (full view in Fig. 4a and enlarged detail in Fig. 4c), completely removed by the NEW algorithm (full view in Fig. 4b and enlarged detail in Fig. 4d).

**Fig. 5:**
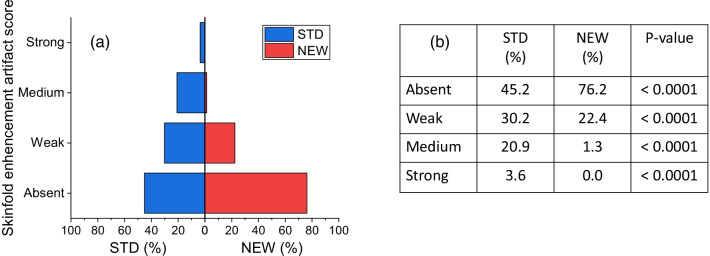
**a** Mirror bar graphs showing the percentages of DES CEM images for which the intensity of skinfold enhancement was scored “Absent”, “Weak”, “Medium” or “Strong” by the majority of readers, when the images were obtained with the STD (blue bars) and the NEW (red bars) algorithm, respectively; **b** results of comparison between proportions associated with STD and NEW algorithms for each category of skinfold enhancement artifact and related *P* values

Although the presence of skinfolds should be classified more an image “defect” due to an incorrect breast positioning than a real artifact, the STD CEM dual-energy subtraction enhances the visibility of possible skin folds; the NEW algorithm also reduces the skinfold enhancement as illustrated in Fig. 5.

**Fig. 6:**
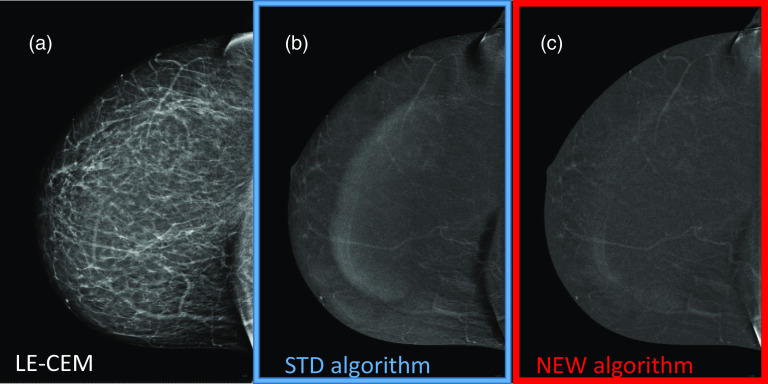
Postmenopausal woman 60 yo at intermediate risk for breast cancer with large, fatty breasts. **a** RCC LE-CEM view showing a large skinfold close to the chest wall; **b** RCC DES CEM view obtained by the STD algorithm showing the skinfold enhancement artifact (in addition to the breast-in-breast artifact); **c** RCC DES CEM view obtained by the NEW algorithm, where the skinfold enhancement is markedly reduced

Skinfold enhancement was detected by the majority of readers in 54.8% of DES CEM images processed with the STD algorithm; such enhancement was systematically either reduced or eliminated by the NEW algorithm. An example of skinfold enhancement reduction is depicted in Fig. 6.

**Fig. 7:**
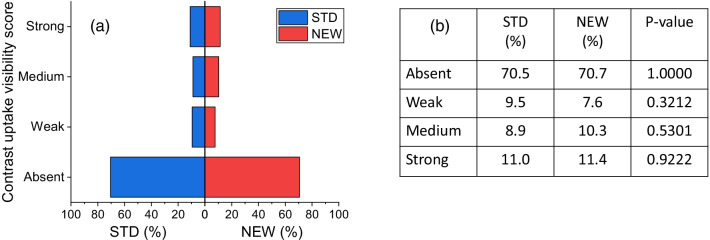
**a** Mirror bar graphs showing the percentages of DES CEM images for which the intensity of contrast uptake visibility was scored “Absent”, “Weak”, “Medium” or “Strong” by the majority of readers, when the images were obtained with the STD (blue bars) and the NEW (red bars) algorithm, respectively; **b** results of comparison between proportions associated with STD and NEW algorithms for each category of contrast uptake visibility and related p-values

The capability of the NEW algorithm of preserving possible contrast uptake visibility associated with lesion presence, along with its effectiveness in reducing or removing the DES CEM artifacts is summarized in Fig. 7.

The proportion of images without contrast uptake was prevalent; this is consistent with the study population focused on the evaluation of CEM performance in the surveillance of women at increased risk for breast cancer. However, it was not found any significant difference in the classification of contrast uptake visibility for images showing lesion contrast enhancement.

**Fig. 8:**
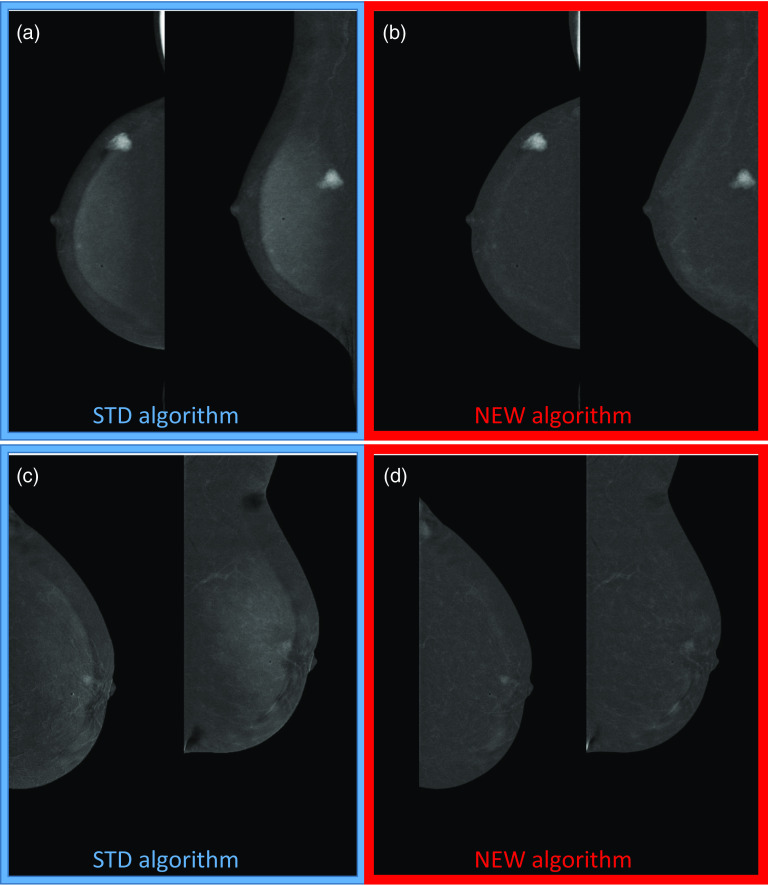
**a** and **b** Postmenopausal woman 55 yo at intermediate risk for breast cancer with small, heterogeneously dense breasts and intense contrast uptake corresponding to a 15 mm lesion in the upper-inner quadrant which was proven to be a high grade invasive lobular carcinoma: **a** RCC and RMLO DES CEM views obtained by the STD algorithm showing a marked breast-in-breast artifact and an intense contrast uptake; **b** RCC and RMLO DES CEM views obtained by the NEW algorithm without artifacts and with the same contrast uptake. **c** and **d** Postmenopausal woman 62 yo woman at intermediate risk for breast cancer with small, heterogeneously dense breasts, showing a tiny contrast uptake corresponding to a 7 mm round lesion behind the nipple: **c** LCC and LMLO DES CEM views obtained by the STD algorithm, with the breast-in-breast artifact partially overlapped to the lesion; **d** LCC and LMLO DES CEM views obtained by the NEW algorithm, where the artifact is eliminated while the lesion is more easily detectable

Figure 8 shows two examples of DES CEM cases with contrast uptake associated with the presence of two breast lesions, where the lesion contrast enhancement was preserved even after artifact suppression by the NEW algorithm.

Table 3 shows for each artifact type the inter-reader agreement in terms of artifact occurrence with the STD algorithm and the percentage of artifact removal and reduction by the NEW algorithm. The Fleiss’ kappa coefficients obtained from the scores assigned by the four readers to each image in the two datasets (same images processed with the STD and the NEW algorithms are also reported as overall agreement metric.

**Table 3:** Inter-reader agreement: Fleiss’ kappa coefficients of the scores assigned to the intensity of breast-in-breast, ripple, and skinfold enhancement artifacts, and to the contrast uptake visibility for the images processed with the STD and the NEW algorithm

	Fleiss’ kappa	
	STD algorithm	NEW algorithm
Breast-in-breast artifact	0.394	0.243
Ripple artifact	0.290	0.249
Skinfold enhancement artifact	0.219	0.328
Contrast uptake visibility	0.458	0.445

The overall agreement was fair (between 0.20 and 0.40) for the artifact assessment and moderate for the contrast uptake visibility assessment (between 0.40 and 0.60).

**Fig. 9:**
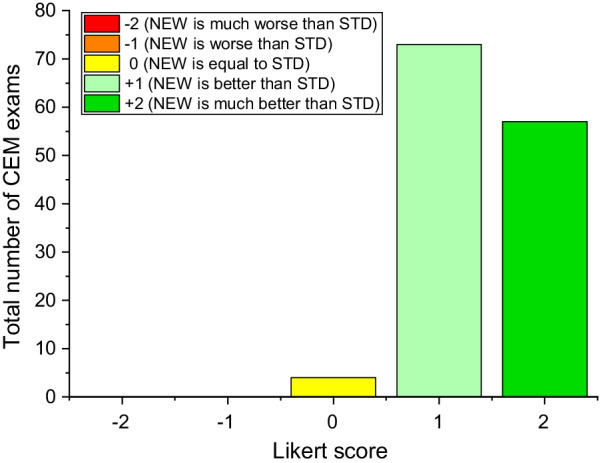
Likert scores by the majority of readers used to compare the overall preferences between the two algorithms. The readers rated the image quality of the CEM images processed by the NEW dual-energy subtraction algorithm “better” or “much better” than images processed by the STD algorithm in 97.8% of cases, while remaining 2.8% of cases were judged “equal” with the two algorithms

Finally, considering the overall per-case comparison between the two algorithms, the preferences by the majority of readers based on the Likert scale are represented in Fig. 9.

In 97.8% of cases the majority of readers considered DES CEM images processed with the NEW algorithm better or much better than those processed with the STD algorithm. Remaining 2.8% of cases (4 out of 134 total cases) were judged “equal” with the two algorithms.

## Discussion

In the dataset considered in this study, the most frequent artifact when dual-energy subtraction images were obtained by the STD algorithm was “breast-in-breast”, occurring in 78.3% of images, followed by the “ripple” and the skinfold enhancement artifacts, occurring with comparable frequency (ripple: 54.2%; skinfold enhancement: 54.8%). The breast-in-breast artifact occurred more frequently in CC views (CC: 55.1%, MLO: 44.9%, *P* = 0.0034), while the ripple and the skinfold enhancement artifacts were both prevalent in MLO views (CC: 40%. MLO: 60%, *P* < 0.0001). The NEW dual-energy subtraction algorithm succeeded in completely eliminating 84.5% of breast-in-breast, 84.2% of ripple, and 56.9% of skinfold enhancement artifacts, while reducing additional 12.1% breast-in-breast, 13.0% ripple and 28.8% skinfold enhancement artifacts. Only a few percent of breast-in-breast and ripple artifacts were not reduced by the NEW algorithm, mostly in images for which the artifacts were rated “Weak” with the STD processing; skinfold enhancement artifacts were not reduced in 14.2% of cases. Importantly, contrast uptake by possible lesions was unchanged when moving from the STD to the NEW algorithm, showing that the NEW algorithm reduces the artifacts while preserving contrast uptake. Overall the NEW algorithm was very effective in reducing or eliminating the DES CEM artifacts without reducing contrast uptake, as confirmed by the readers’ preferences through the Likert scores: 131 out of 134 cases processed with the NEW algorithm were rated better or much better than those processed with the STD algorithm. This shows that the NEW algorithm clearly improved the DES CEM image quality, increasing readers’ confidence.

In a retrospective analysis by Yagil et al. the authors reported that the breast-in-breast artifact was found in 98% of subtraction images (both cranio-caudal, CC, and medio-lateral oblique, MLO), while the ripple artifact was detected in 32% of subtraction images, predominantly MLO views; however, none of the artifacts interfered with image interpretation [9]. Most of the few papers on CEM-related artifacts are pictorial reviews, describing the different artifact types, and “lightly” assessing that usually the artifact presence does not impact on interpretation [6, 7]. Nevertheless, the potential impact of artifacts in CEM image interpretation could be more or less relevant, depending on the clinical task and on the lesion type and size. For example, when CEM is used as a detection tool, as in the screening of women at increased risk for breast cancer, the presence of artifacts might disturb the detectability of small lesions. Moreover, as reported by Kamal et al. 30% of breast cancers found with CEM were non-mass lesions, and that most malignant lesions in CEM show faint contrast uptake [16]. This suggests that possible artifact presence might compromise or at least reduce the visibility of those faint findings.

Another clinical factor which could be affected by the presence of artifacts is the background parenchymal enhancement (BPE) classification [17], that is emerging as a potential risk factor [18], and is sometimes used for decisions such as the choice of modality for image-guided biopsy.

In general, clinical assessment of new imaging technologies is performed step-by-step. Once the clinical effectiveness of the new technology versus a reference standard is demonstrated, the following step while this new imaging technique is more and more applied in clinical practice is to optimize image quality while reducing/removing artifacts. Statements about a “presumed absence of CEM artifact impact” are highly subjective and insufficient for clinical application, without supporting results. This study has shown that it is possible to develop dual-energy subtraction algorithms able to reduce CEM-related artifacts. Ideally, it would be desirable if the optimization of new imaging technologies by manufacturers would be started well before such technology becomes widely used in clinical practice. Nevertheless, as remarked by Neppalli et al., it is fundamental to store unprocessed DICOM images into the PACS to enable image reprocessing in case of new algorithm developments or improvements, as occurred for the present study [8].

The image quality optimization effort from CEM manufacturers is equally important as the effort which should be made by the CEM users to standardize the clinical protocols and the image interpretation lexicon, as recommended by Sardanelli et al. in a critical review [19].

This study has limitations: it was a side-by-side comparison using a qualitative scale with limited inter-reader agreement (as shown by the Fleiss’ kappa coefficients), the sample size was relatively small and all the CEM mammography were performed at a single center with a unique equipment.

Future research should consider if the BPE classification might be affected by CEM-related artifact presence, and better investigate the relationship between each type of artifact and characteristics of images, breasts and positioning.

## Conclusion

The new dual-energy subtraction algorithm evaluated in this study demonstrated to be effective in dramatically reducing or eliminating CEM-related artifacts, while preserving lesion contrast enhancement.

## Data Availability

The datasets used and/or analysed during the current study are available from the corresponding author on reasonable request.

## Abbreviations

BPE: Background parenchymal enhancement
CC: Cranio-caudal (view)
CEM: Contrast-enhanced mammography
DES: Dual-energy subtraction
HE: High-energy
LE: Low-energy
MLO: Medio-lateral oblique
NEW: New DES algorithm suppressing CEM artifacts
STD: Standard DES algorithm
